# Implementation of central venous access device care bundles using a plan-do-study-act approach to reduce central line-associated bloodstream infection in children at a tertiary hospital in Indonesia

**DOI:** 10.3389/fped.2026.1718071

**Published:** 2026-04-10

**Authors:** Yogi Prawira, Nina Dwi Putri, Dwi Putri Lestari, Pramita Gayatri Dwipoerwantoro, Irene Yuniar, Aryono Hendarto, Idham Jaya Ganda

**Affiliations:** 1 Department of Pediatrics, Faculty of Medicine, Universitas Indonesia, Dr. Cipto Mangunkusumo National Central Hospital, Jakarta, Indonesia; 2 Department of Pediatrics, Wahidin Sudirohusodo Hospital, Makassar, Indonesia

**Keywords:** critical care, infection control, pediatrics, Plan-Do-Study-Act (PDSA), quality improvement

## Abstract

**Background:**

Sepsis is a major cause of morbidity and mortality worldwide, especially in low- and middle-income countries. Central line-associated bloodstream infection (CLABSI) contributes significantly to hospital-onset sepsis. However, data on CLABSI rates related to central venous catheter placement and bundle non-compliance in our hospital are limited.

**Aim:**

To evaluate a Plan-Do-Study-Act (PDSA) based CLABSI bundle in reducing CLABSI rates in children at Dr. Cipto Mangunkusumo Hospital.

**Methods:**

A quality improvement study using three sequential PDSA cycles was conducted between September and November 2022. Children aged 1 month to 18 years who underwent central venous access device (CVAD) insertion by the Pediatric Emergency and Intensive Care team between June and November 2022 were included. Interventions comprised education on the CLABSI bundle and CVAD insertion practices, increased nursing involvement through standardized bedside training, and reinforced documentation and monitoring of CVAD care.

**Results:**

A total of 280 patients were included, with 143 in the pre-intervention period and 137 during the intervention period. The mean CLABSI rate decreased from 12.7 to 8.6 per 1,000 central-line days within three months of PDSA implementation. The lowest CLABSI rate, 3.4 per 1,000 central-line days, was observed in November 2022. Process evaluation PDSA Cycle 3 identified persistent system-level barriers, including limited availability of essential supplies and suboptimal adherence to recommended insertion and dressing practices.

**Conclusion:**

PDSA-based CLABSI bundle implementation was feasible and associated with an early reduction in CLABSI rates; however, system-level and resource constraints limited sustained improvement.

## 1 Introduction

Sepsis continues to be a major cause of morbidity and mortality worldwide, despite improvements in medical care and increased awareness. The incidence rate of sepsis is 677.5 cases per 100,000 population worldwide and it is responsible for > 8% of all pediatric intensive care unit (PICU) admissions (1, 2). Children in developing countries have a significantly higher sepsis-related mortality rate compared to those in developed countries (31.7% vs. 19.3%) (3). In addition, hospital-onset sepsis is associated with approximately a twofold higher odds of death compared to community-onset sepsis (4).

A central line-associated bloodstream infection (CLABSI) is a laboratory-confirmed bloodstream infection (LCBI) that is not secondary to an infection at another body site, such as urinary tract infection (UTI), pneumonia, or surgical site infection. In this study, central venous access devices (CVADs) included centrally inserted central catheters (CICCs) and femorally inserted central catheters (FICCs), both of which are non-tunneled intravascular catheters with their tips positioned in a central vein and represent the standard CVADs used by the Pediatric Emergency and Intensive Care team in our institution. CLABSIs are considered the most preventable type of healthcare-associated infection (HAI) (5–7). As reported by the International Nosocomial Infection Control Consortium (INICC), CLABSI rates are significantly higher in low- and middle-income countries (LMICs) than in high-income countries. CLABSI rates in resource-limited countries range from 1.6 to 44.6 per 1,000 central-line (CL) days in adult and pediatric ICUs, and 2.6 to 60 per 1,000 CL days in the neonatal intensive care unit (NICU) (7). The incidence of CLABSIs in the perinatology unit at RSCM before 2015 was 8.7%. After the implementation of the CLABSI bundle, the incidence of CLABSIs from 2016 to 2018 remained fluctuating, while the proportion of neonatal patients who died with CLABSIs increased to 57.7% in 2018 (8). Research conducted in a hospital in Taiwan from 2015 to 2017 found that adherence to PDSA interventions reduced the CLABSI rate by 59.5% and led to a reduction in the average duration of central venous catheter use in children and neonatal patients treated in PICU and NICU (9).

To the best of our knowledge, there is currently no data regarding CLABSI rates associated with central venous catheter placement and adherence to CLABSI bundles at Department of Pediatrics, Dr. Cipto Mangunkusumo Hospital, Jakarta, Indonesia. In Indonesia, existing studies have primarily focused on neonatal settings. A meta-analysis reported variable CLABSI incidence associated with umbilical venous catheter (UVC) and peripherally inserted central catheter (PICC) use in preterm infants, emphasizing the burden of catheter-related infections locally (10). Risk factor studies from tertiary NICUs also identified catheter duration and insertion site as key predictors of CLABSI (11, 12). However, evidence on bundle compliance or structured quality improvement initiatives in children remains limited, highlighting the need for this study.

## 2 Methods

### 2.1 Study population

Children aged from 1 month to 18 years who were indicated for a central venous catheter procedure were recruited for the study. Exclusion criteria included all individuals aged less than 1 month or individuals aged 1 month to 18 years who had previously undergone central venous catheter placement carried out by other than the Dr. Cipto Mangunkusumo Hospital Pediatric Emergency and Critical Care team, including teaching staff, all students in subspecialist education programs and fellowships in the Pediatric Emergency and Critical Care division.

### 2.2 Quality improvement intervention

The quality improvement intervention focused on the hospital's CLABSI prevention bundles. The team consisted of the head nursing staff from the pediatric intensive care unit (PICU) and the cardiac intensive care unit (CICU), the infection prevention and control nurse (IPCN), the infection prevention and control doctor (IPCD), and the infection control consultant. The study was conducted at Dr. Cipto Mangunkusumo Hospital, an academic tertiary hospital in Jakarta, Indonesia, from June 2022 to November 2022. All procedures were performed in the emergency room, the PICU, and the CICU.

### 2.3 Outcome and process measures

The team developed the PDSA bundle as an intervention on the best available evidence from the literature, previous experience, and a previously published conceptual model. Given that this was a single-center study conducted in a tertiary hospital in LMIC, where healthcare systems are often complex and resource constraints may affect implementation, the PDSA method was considered suitable to support adaptive and multidisciplinary intervention refinement. Monthly meetings were held during the period September 2022–November 2022 to review and analyze the results of the intervention in each cycle. The team determined 3.5 per 1,000 central-line days to be the minimum target for the monthly CLABSI rates as the outcome measure, while the target of CLABSI bundle non-compliance to be 0% as the process measure.

### 2.4 Data analysis

CLABSI rates were analyzed for the three months prior to Plan-Do-Study-Act (PDSA) implementation and the three months following implementation. The data were analyzed using IBM SPSS Statistics 26. The monthly CLABSI rates were presented in a run chart to support process monitoring and signal detection over time. The control charts were generated using the plug-in QI Macros for Microsoft Excel 2016.

#### 2.4.1 PDSA cycle 1 (September, 1st 2022 – September, 30th 2022)

The initial intervention aimed to improve healthcare workers' knowledge of the CLABSI bundle and recommended CVAD insertion practices. Our team provided formal education and training on CLABSI bundle prevention for the nurses, medical staff, and fellowship students from the pediatric emergency and critical care department who possessed the competencies for CVAD insertion procedures or care, through both offline and online training courses.

After reviewing the results from the first PDSA cycle, the team observed an increase in CLABSI rates. The team discovered that there was still a shortfall in the healthcare workers' compliance with utilizing complete CVAD kits. There was also non-compliance among healthcare professional in choosing a CVAD insertion site based on the recommended practice, which they attributed to several variables, including operator skill, mechanical issues, and relative contraindications (coagulopathy, thrombocytopenia).

#### 2.4.2 PDSA cycle 2 (October, 1st 2022 – October, 31st 2022)

The main focus in PDSA cycle 2 was to increase pediatric nursing participation in the CVAD care bundle to prevent CLABSI in children, and the initial plan from PDSA cycle 1 was abandoned. The team consulted with specialists and also held a coordination with the head nurse and nursing staffs to design the standard practice for CVAD insertion. Afterwards, the team conducted bedside teaching sessions and trained nurses according to the standard practice.

Process measures during this cycle remained fluctuating. Non-compliance with CVAD site selection and CVAD care remained high, while non-compliance related to CVAD equipment decreased. The head of the nursing unit established a specialized care unit, equipped with the emergency trolleys and a vital signs monitor device, dedicated to the CVAD insertion process. The area was also equipped with CVAD disposable kits, which included sterile surgical drapes and needle holders.

#### 2.4.3 PDSA cycle 3 (November, 1st 2022 – November, 30th 2022)

The outcome showed a decrease in CLABSI rates following the PDSA cycle; thus, the team decided to adapt and continue the intervention while monitoring progress in PDSA cycle 3. Nurses were also encouraged by the team to fill the daily hospital acquired infection form and maintain complete records of CVAD care. However, during the process, the team identified persistent gaps, including frequent unavailability of sterile disposable drapes, frequent use of gauze dressings instead of transparent dressings, and non-compliance with recommended CVAD insertion site selection.

## 3 Results

### 3.1 Patient characteristics

The total number of subjects participating in this study was 280 patients, with 143 subjects during the pre-intervention period and 137 subjects in the intervention period (Table 1). Based on the data from the pre-intervention period and post-intervention periods, children with CLABSI were predominantly male (80.0% during the pre-intervention period; 58.3% during the post-intervention period). Most patients admitted with a medical diagnosis (85.0% in pre-intervention period; 91.7% in the post-intervention period) had CLABSI more frequently than those with surgical diagnosis. Based on the setting where CVAD was inserted, most CLABSI cases occurred in the medical ward in the pre-intervention period (50.0%), and in both the medical ward (41.6%) and the PICU (41.6%) during the post-intervention period. Patients with CVAD insertion via the femoral vein (66.7%) had a higher proportion of CLABSI compared with those with internal jugular vein insertion in the post-intervention period, while during the pre-intervention period, both insertion sites had an equal number of CLABSI cases. Children with CLABSI had a longer median duration of CVAD placement compared with children without CLABSI in both the pre-intervention and post-intervention periods. However, children with CLABSI had a shorter median length of stay in the pre-intervention period (18.5 vs. 21.0), but children with CLABSI in the post-intervention period had a longer median length of stay than children without CLABSI (32.0 vs. 18.0).

**Table 1:** The demographic data.

Characteristics	Pre-intervention		Post-intervention	
	Non—CLABSI *N* = 123	CLABSI *N* = 20	Non –CLABSI *N* = 125	CLABSI *N* = 12
Age (month) median (range)	27.0 (1–206)	20.5 (7–206)	47.9 (1–214)	18.5 (2–212)
Sex, *n* (%)				
Male	60 (48.8)	16 (80.0)	59 (47.2)	7 (58.3)
Female	63 (51.2)	4 (20.0)	66 (52.8)	5 (41.7)
Diagnosis categorization, *n* (%)				
Medical	108 (87.8)	17 (85.0)	116 (92.8)	11 (91.7)
* Critically ill*	83	9	78	7
* Non Critically ill*	25	8	38	4
Surgical	15 (12.2)	3 (15.0)	9 (7.2)	1 (8.3)
CVAD insertion setting, *n* (%)				
Emergency ward	57 (46.3)	6 (30.0)	48 (38.4)	2 (16.7)
PICU	35 (28.5)	4 (20.0)	35 (28.0)	5 (41.6)
Medical ward	31 (25.2)	10 (50.0)	42 (33.6)	5 (41.6)
Insertion site, *n* (%)				
Femoral vein	78 (63.4)	10 (50.0)	89 (71.2)	8 (66.7)
Internal jugular vein	45 (36.6)	10 (50.0)	36 (28.8)	4 (33.3)
CVAD placement duration, median (range)	9.0 (1–41)	9.5 (2–55)	11.0 (1–53)	17.5 (8–39)
Length of stay, median (range)	21.0 (1–114)	18.5 (2–104)	18.0 (1–95)	32.0 (11–45)

### 3.2 CLABSI rate trends

**Figure 1:**
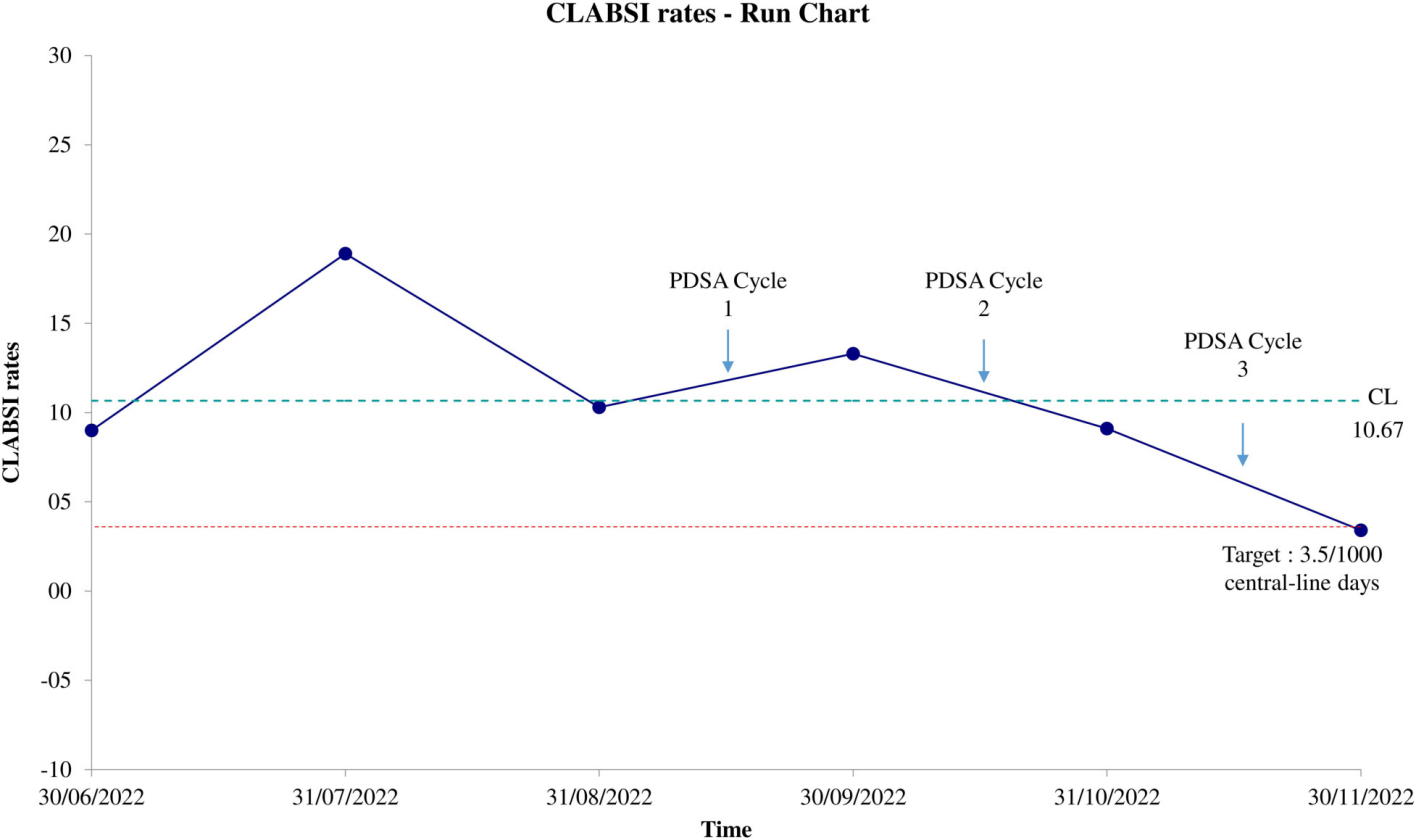
CLABSI run-chart.

From June 2022 to November 2022, the team noted a decrease in CLABSI rates of 4.1 per 1,000 central-line days, from an average of 12.7 per 1,000 central-line days during the pre-intervention period (June 2022 to August 2022) to an average of 8.6 per 1,000 central-line days during the intervention period (mid-September 2022 to November 2022). Although there was a decrease in CLABSI rates, the rates during the intervention period were not significantly lower when compared with the pre-intervention period (*p* = 0.384). Based on the run chart, CLABSI rates showed a downward trend (five consecutive descending) after July 2022, and reached the CLABSI rate of 3.4 per 1,000 central-line days by the end of the study, which is below our determined target of 3.5 per 1,000 central-line days as shown in Figure 1 and Table 2.

**Table 2:** CLABSI rates (per 1,000 central-line days).

Month	Length of central-line days	CLABSI	CLABSI per 1,000 central-line days
June	663	6	9
July	529	10	18.9
August	388	4	10.3
September	448	6	13.4
October	437	4	9.1
November	577	2	3.4

### 3.3 Bundle compliance

**Figure 2:**
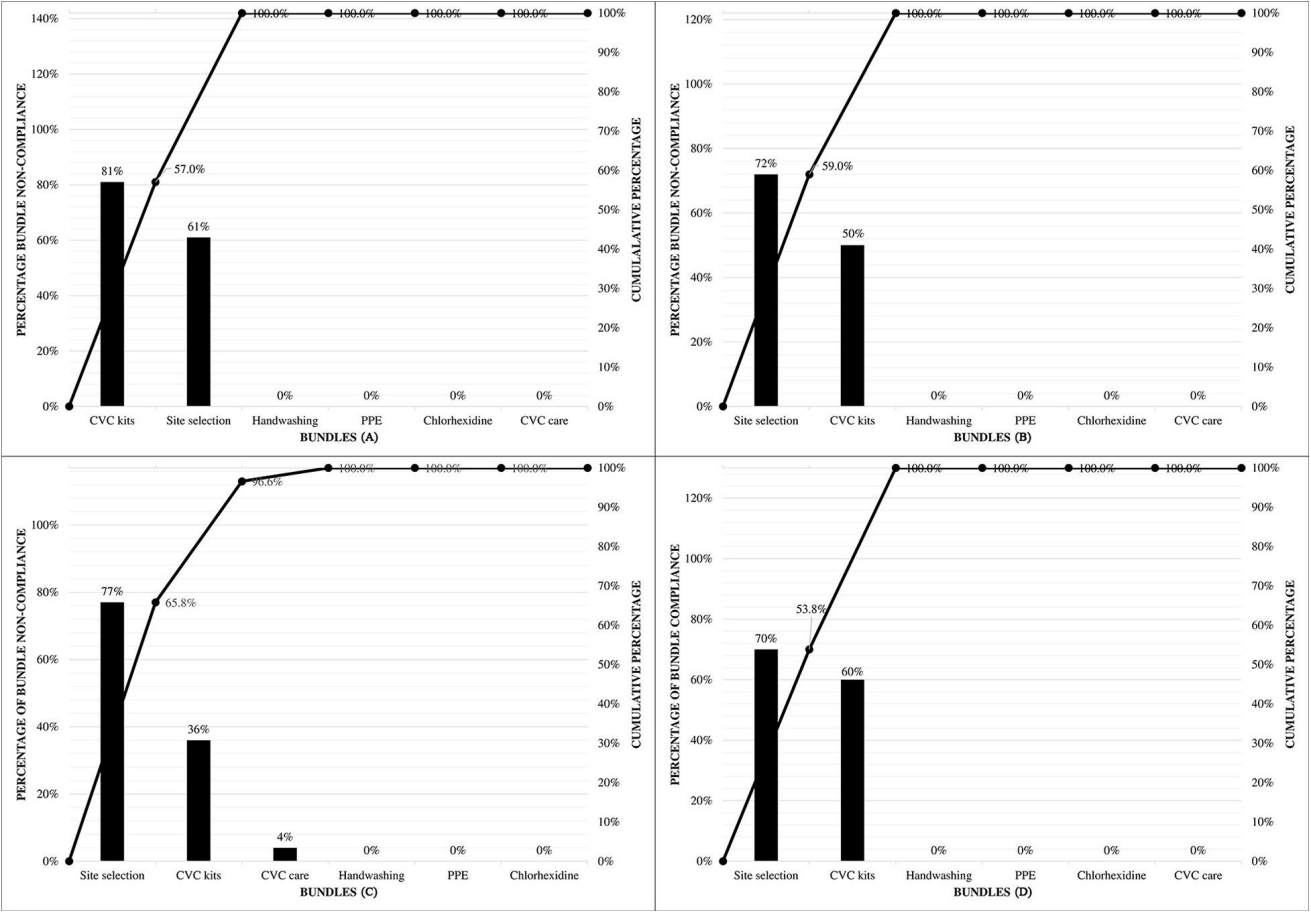
Pareto chart illustrating bundle non-compliance during the pre-intervention period **(A)**, after PDSA cycle 1 **(B)**, after PDSA cycle 2 **(C)**, and after PDSA cycle 3 **(D)**. Before the intervention **(A)**, most non-compliance was due to incomplete CVAD kits and inappropriate site selection. After Cycle 1 **(B)**, kit-related issues improved, but site selection remained the main problem. In Cycle 2 **(C)**, overall non-compliance decreased further, including improvements in insertion and care practices. By Cycle 3 **(D)**, most kit and care issues were resolved, although site selection continued to be the largest remaining source of non-compliance.

The process measures assessed healthcare workers' compliance with the CLABSI bundle. Two CLABSI bundles did not achieve the target, namely CVAD kits and site selection, while handwashing, chlorhexidine, PPE and CVAD care achieve zero percent bundle non-compliance as shown using Pareto chart in Figure 2. The CVAD kits bundle non-compliance percentage at the end of the study (60%) was lower than that in the pre-intervention period (81%), while the site selection bundle non-compliance percentage at the end of the study (70%) was higher than that in the pre-intervention period (61%).

## 4 Discussion

This study suggests that the PDSA approach was associated with a downward trend in CLABSI rates. The highest CLABSI rate was recorded in July 2022, while the lowest CLABSI rate occurred in November 2022, with two peaks noted in July and September 2022. CLABSI rates reached the targeted level by the end of the study period.

Sustained compliance was observed in four bundle indicators (hand washing, PPE, chlorhexidine use, and CVAD care). Meanwhile, two indicators (CVAD kits and site selection) did not achieve the target at any time point. Despite the discrepancy, a reduction in CLABSI rates was observed, suggesting that improvement in CVAD maintenance care and nursing engagement may have a greater impact on reducing CLABSI than insertion-related factors in this study.

This study is aligned with previous studies showing that male sex predominated in the CLABSI group in patients with CVAD (13, 14). Patients admitted with medical diagnosis had a higher rate of CLABSI, which was also found in another study (15), although others have reported higher rates in surgical patients possibly due to varying numbers of cases or sample size (16). In our study, longer CVAD placement duration in the CLABSI groups was consistent with findings from previous studies, which show that CLABSI groups had longer CVAD duration compared to the non-CLABSI group (13, 14). Some studies indicated that prolonged CVAD placement duration may result in CLABSI due to the formation of biofilms. These biofilms tend to form on the external lumen of the catheter device, but after 10 days, the biofilms may go inside the lumen of the catheter and cause CLABSI (17, 18).

Over the course of the study, the two peak CLABSI rates may have been associated with the arrival of new trainee fellows or subspecialty students who were still undergoing mentorship and training in CVAD insertion procedures. However, the team mitigated this issue by providing proper training regimens. The four bundle practices—hand washing, PPE, the use of chlorhexidine, and CVAD care—achieved zero percent non-compliance at the end of the pilot study. The use of transparent dressing allows easier visibility of the catheter exit site compared to gauze dressings and may help prevent bacterial translocation. Previous studies have shown that the use of transparent dressings can increase catheter dwell time and reduce the incidence of complications (19). These practices were also highly feasible, as they were supported by our hospital's long-existing policy, which has been adhered to for many years and is based on the standardized national Indonesian Hospital Ministry policy (20).

Although the result showed that two bundle non-compliances (CVAD kits and site selection) did not significantly improve, CLABSI rates still tended to decline following the intervention, which may be attributed to the team's direct participation and monitoring during the procedure, as well as persistent effort to enforce bundle compliance. The team hypothesized that additional practices and increased nursing participation in CVAD care were major factors in reducing CLABSI incidence in our department. A similar study showed that intervention involving nursing participation and CVAD maintenance care had a more significant impact in reducing CLABSI rates from 4.00 per 1,000 central line days at baseline to 1.81 per 1,000 central line days than interventions involving on CVAD site selection (21). Another study showed that direct nursing interventions in CVAD maintenance care reduced CLABSI rates from 2.25 per 1,000 central line days at baseline to 1.79 per 1,000 central-line days in pediatric oncology patients (22).

A quality improvement study mentioned earlier from King Abdulaziz Hospital in Saudi Arabia reduced CLABSI rates from an average of 2.0 per 1,000 central-line days in 2008 to 0.7 per 1,000 central-line days in 2010 using PDSA as a quality-improvement method. The difference between the Saudi study and ours was that their study was able to maximize the overall compliance bundle level up to 100% at the end of the study (23). Nonetheless, one study reported that there was a significant difference between bundle compliance and the improvement in CLABSI rates with PDSA intervention. The results showed that even though compliance with the bundle of interventions was high, the improvement in CLABSI rates was not as expected. The study also highlighted the importance of continuous monitoring and evaluation to ensure intervention effectiveness in achieving the desired outcomes (24). These findings emphasize the importance of continuous monitoring, adaptation, and prioritization, particularly in resource-constrained environments.

The team also faced clinical decision-making challenges, such as balancing the advantages and disadvantages of vein selection while managing infection risk and patient comfort (25). Previous studies have suggested that upper body vein selection reduces the risk of infection in CVAD procedures (6). On the other hand, recent evidence has shown no significant difference in bloodstream infection rates among the three central veins, suggesting that the low compliance with the site-selection bundle in our study did not affect the CLABSI rates (26). Additionally, the lack of CVAD procedure guidelines in children may contribute to the low compliance with site selection. During the study, the hospital adhered to CVAD procedure guidelines based on the study of adult population studies, which may have created a clinical practice gap between adult and children.

Although the PDSA method is relatively simple to understand, its implementation remains challenging. The relatively short study period and insufficient monitoring and evaluation data following the intervention period represent a key limitation of this study, as the PDSA method requires an ongoing process and a commitment to be effective in its implementation. As this was a single-center study, the finding may not be generalizable to other healthcare settings with different resources, staffing, or patient populations.

A possible confounding factor, such as the arrival of new trainee fellows or subspecialty students during the study period, may have affected the data on CLABSI rates and on the analysis, due to variability in skill levels and procedural familiarity. This staff turnover highlights the need for structured and continuous training. This study also had several limitations, including the lack of an adequate supply of CVAD kits, which was highly dependent on hospital policy and financial constraints, and the absence of standardized CVAD procedure and care guidelines which was intended specifically for children were not yet established in our institution. These structural barriers limited compliance with CVAD bundle elements and highlight the necessity for stronger engagement and support from the health authorities and policymakers to advance quality improvement study in reducing CLABSI through comprehensive evaluation and intervention. As previously noted, the application of PDSA requires substantial preparation in leadership, knowledge, and resources (27).

## 5 Conclusion

Implementation of a PDSA-based CLABSI prevention bundle was feasible and associated with an early reduction in CLABSI rates, decreasing from 12.7 to 8.6 per 1,000 central-line days and reaching 3.4 per 1,000 central-line days by November 2022. These improvements were associated with strengthened maintenance practices and greater adherence to CVAD maintenance protocols.

However, findings from PDSA Cycle 3 highlighted persistent system-level barriers, including limited availability of essential supplies, inconsistent adherence to recommended insertion and dressing practices, and operator-dependent variability that constrained sustained improvement. Continued progress will require reliable resource allocation, strengthened procedural standardization, and ongoing competency-based training to support durable CLABSI reduction in pediatric critical care settings.

## Data Availability

The original contributions presented in the study are included in the article/Supplementary Material, further inquiries can be directed to the corresponding author.
